# Optic Nerve Cysticercosis at the Orbital Apex

**DOI:** 10.18502/jovr.v15i1.5965

**Published:** 2020-02-02

**Authors:** Mahmood Dhahir Al-Mendalawi

**Affiliations:** ^1^Department of Paediatrics, Al-Kindy College of Medicine, University of Baghdad, Baghdad, Iraq

Dear Editor,

I read with interest the case report by Goel^[1]^ published in the October–December 2018 issue of the *Journal of Ophthalmic and Vision Research*. The author described nicely a case of optic nerve cysticercosis at the orbital apex presenting as optic neuritis in an Indian patient.^[1]^ It is well-known that due to compromised immune system, individuals infected with human immunodeficiency virus (HIV) are more susceptible to various types of viral, bacterial, fungal, and parasitic infections compared to the individuals with healthy immune system. Among parasitic infections, cysticercosis has been reported among HIV-positive patients.^[2]^ To my knowledge, HIV infection is a distressing health issue in India. The available data pointed out to 0.26% HIV seroprevalence compared with a global average of 0.2%,^[3]^ and the overall seropositivity for cysticercosis was reported to be 5% among HIV-positive patients in India.^[4]^ I assume that the underlying HIV infection ought to be taken into consideration in the studied patient. Accordingly, planning for the diagnostic battery of blood CD4 count and viral overload measurements for HIV infection was envisaged. If that battery was contemplated and it disclosed HIV reactivity, the case in question could be obviously regarded the second novel case report of HIV-associated orbital cysticercosis in India. The first report was of subretinal cysticercosis in an Indian patient with AIDS reported nearly two decades ago.^[5]^

##  Financial Support and Sponsorship

Nil.

##  Conflicts of Interest

There are no conflicts of interest.
